# Complete genome sequence of the *Stenotrophomonas* sp. KACC 24087 and *Parapusillimonas* sp. KACC 24088, isolated from livestock wastewater treatment

**DOI:** 10.1128/mra.00955-25

**Published:** 2025-11-17

**Authors:** Jae-Cheol Lee, Hyorim Choi, Heisu Kim, Seunghwan Kim, Yiseul Kim, Jun Heo

**Affiliations:** 1 Department of Environmental Engineering, School of Architecture, Civil and Environmental Engineering, Mokpo National University 34991, Muan, Republic of Korea; 2 Agricultural Microbiology Division, National Institute of Agricultural Sciences, Rural Development Administration 230986, Wanju-gun, Jeollabuk-do, South Korea; University of California Riverside, Riverside, California, USA

**Keywords:** *Stenotrophomonas*, *Parapusillimonas*, genome, novel species

## Abstract

We report the whole genome sequence of two species. Both strains were isolated from the influent and effluent of a wastewater treatment plant and are expected to contribute to the understanding of microbial diversity in the environment and the discovery of useful microbial resources.

## ANNOUNCEMENT

Livestock wastewater contains high concentrations of organic matter and nitrogen, and diverse microorganisms are commonly detected in its treatment systems (1, 2). From the influent of a livestock wastewater treatment facility in Muan-gun, Republic of Korea, we isolated strains in August 2024 from the effluent of lab-scale, cold plasma-based advanced oxidation process (AOP) reactor (3). The reactor surface was swabbed with a sterile cotton swab, and the collected sample was streaked onto R2A agar plates. Random colonies were subsequently selected and sub-cultured to obtain single isolates. The 16S rRNA sequencing was conducted for each isolate. Among them, two strains were identified that were distinguishable from other species.

We report the whole-genome sequences of *Stenotrophomonas* sp. KACC 24087 (= JC08) and *Parapusillimonas* sp. KACC 24088 (= JC17), it will enhance understanding of microbial community shifts during treatment and provide insights into their responses under oxidative conditions (4).

For the extraction of DNA, the strains were cultured on R2A medium (BD Difco, NJ, USA) at 28°C for 3 days under aerobic conditions. The Qiagen MagAttract HMW DNA kit (Qiagen, Hilden, Germany) was used for genomic DNA extraction. DNA concentration was measured using PicoGreen, and size distribution was assessed using the Agilent Femto Pulse (Agilent, CA, USA). Genomic DNA was sheared using the Megaruptor 3 (Diagenode, NJ, USA) and purified using AMPure PB beads. For PacBio sequencing, a total of 10 µL library was prepared using the SMRTbell Prep Kit 3.0 (Pacific Biosciences, CA, USA). SMRTbell templates were annealed using the Revio SPRQ polymerase kit and sequenced on the PacBio Revio platform by Macrogen (Korea). PacBio subreads were assembled using the microbial assembly application in SMRTLink v25.1.0 based on the Hierarchical Genome Assembly Process 4 (5). Gene prediction was performed using the GeneMarkS-2 +v6.10 (6), and basic annotation was conducted by the NCBI Prokaryotic Genome Annotation Pipeline v6.4 (7). All tools were executed with default parameters unless stated otherwise.

For *Stenotrophomonas* sp. KACC 24087**,** PacBio sequencing generated 68,434 long reads (592,762,956 bp; 9,985 *N_50_* with a sequencing depth of 169.79×). The genome was assembled as a single circular chromosome (3,489,563 bp with GC content of 65.8%) and no plasmid was detected. The final assembly includes 3,121 coding sequences, 11 rRNA genes, 61 tRNA genes, 4 ncRNA genes, and 49 pseudo genes.

For *Parapusillimonas* sp. KACC 24088, the PacBio sequencing generated 86,462 long reads (699,837,401 bp; 9,317 *N_50_* with a sequencing depth of 147.24×). The assembled genome consists of a single circular chromosome (4,750,728 bp with GC content of 61.1%) and no plasmid was detected. The final assembly includes 4,473 total coding sequences, 9 rRNA genes, 49 tRNA genes, 4 ncRNA genes, and 67 pseudogenes.

## Data Availability

The complete genomes of *Stenotrophomona*s sp. KACC 24087 and *Parapusillimonas* sp. KACC 24088 was deposited in NCBI GenBank under the BioProject accession number PRJNA1282443 and PRJNA1282447, the BioSample accession number SAMN49649676 and SAMN49649693, and the GenBank number accession CP197473 and CP197474. The raw PacBio reads can access the NCBI’s SRA accession number SRR34290124 and SRR34290091, respectively.

## AFTER EPUB

*[This article was published on 17 November 2025 with errors in the author byline. The byline was corrected in the current version, posted on 28 November 2025.]*
